# Supplementary material for: QUATgo: Protein quaternary structural attributes predicted by two-stage machine learning approaches with heterogeneous feature encoding
Source: PLoS One. 2020 Apr 29;15(4):e0232087. doi: 10.1371/journal.pone.0232087 (PMC7190164; doi:10.1371/journal.pone.0232087)
Supplement: Attachment — Submitted filename: Response to Reviewer_QUATgo_1015.docx [file pone.0232087.s001.docx]

**Institute of Genomics and Bioinformatics**

**National Chung Hsing University**

**Taichung, Taiwan 40227 ROC**

October 15, 2019

Dr. Mingyang Lu

Academic Editor

PLOS ONE

Dear Dr. Lu,

Thanks for your careful review of my manuscript (PONE-D-19-21078). I really appreciate your help and the comments from both reviewers. After carefully reading the comments raised by the reviewers, I think most comments or problems can be solved. After trying our best to answer all the comments, I would like to resubmit this revised manuscript. All the revised portions were highlighted in yellow color in this revised manuscript. Many thanks for your help. Looking forward to hearing from you.

Sincerely yours,

Yen-Wei Chu

Professor

Institute of Genomics and Bioinformatics

National Chung Hsing University

Taichung, Taiwan 40227 ROC

Tel: 886-4-22840338 ext. 7041

Fax:886-4-22859329

E-mail: ywchu@nchu.edu.tw

**I. Answer to comments from Reviewer #1:**

1. Writing throughout needs work.

Response:

Thank you for your advice, and we appreciate the chance to make this manuscript clear. We have our manuscripts checked by English language native speakers for English editing. The correctness of English writing has been improved. In addition, the English revision certificate has been uploaded as an attachment.

2. The definition of "quaternary structure" repeated many times in introduction.

Some sentences in the introduction are directly copied from Wikipedia.

Response:

Thanks for your comment. According to your suggestion, we have rewritten the first paragraph of the introduction. We restate the definition of quaternary structure of protein and the importance of the quaternary structure prediction research while avoiding plagiarism.

3. Introduction: "In recent years, Shen et al. have also proposed coding methods based on 71 Functional Domain Composition" lacks a citation.

Response: Thank you for reminding. We have added the citation (reference number: [1]) correctly.

**II. Answer to comments from Reviewer #2:**

1. This MS does not show it is superior to existing method. Looking at Table 9 on page 15, for many of the categories, its precision is smaller than that of "QuarIdent".

Response:

Thanks for your comment. Although some results were worse than QuarIdent in precision, the overall average of QUATgo was better than QuarIdent. At the same time, we mainly use MCC as the evaluation index, because it can take into account both true and false positives and negatives and is generally regarded as a balanced measure which can be used even if the classes are of very different sizes. For each independent test, QUATgo was superior to the other two existing methods in MCC results. And, the overall average ACC and MCC results of QUATgo are also higher than the other two methods.

2. Actually, this kind of comparison is meaningless because we do not know many possible overlaps of the testing set with the training set and do not know how similar is the testing set to the training set.

Response:

Thanks for your comment. These two data sets do not have the same or similar protein family. In the homo-oligomers and hetero-oligomers data set, we compare the testing set and the training set where 95% of the sequence data in testing set has sequence identity lower than 40%.

3. I do not even know how the "independent test set" is constructed other than a sentence saying "We compared QUATgo with other quaternary structure prediction tools. We use the remaining files in the training set as independent sets".

Response:

Thank you for your advice, and we appreciate the chance to make this manuscript clear. In revised manuscript, we rewrite two parts of statements as follows.

. Adding the sentence “The remaining data were serve as independent data.” In the section “Flowchart and system construction” to show how the independent test set was made.

. Rewriting the sentences of “We compared QUATgo with other quaternary structure prediction tools by independent test set, and calculated precision, sensitivity, specificity, accuracy (ACC), and MCC.” In the section “Comparing with different prediction tools”

4. The fact that this MS's method is doing well on the larger "MERS" could be due to that there are high sequence identity sequences in the training set to the testing set because for these "MERS", the high sequence identity ones are not removed by CD-hit.

Response:

Thanks for your comment. We further inspected the sequence identity of these MERS S ectodomains to our training set. The results showed that the sequence identity of these hetero-12 mer are about 60%. Please see the table below for detailed results.

| PDBID | Identity |
| --- | --- |
| 5W9H | 62.16% |
| 5W9I | 59.81% |
| 5W9J | 59.81% |
| 5W9K | 60% |
| 5W9L | 59.81% |
| 5W9M | 62.16% |
| 5W9N | 62.16% |
| 5W9O | 62.16% |
| 5W9P | 61.26% |
| 5W9Q | <10% |

5. The MS is written in poor English. There are many sentences like these:

"The quaternary structure of the homo-oligomer can be encoded by the Equation an, where n is the copy number of the polypeptide chain."

"If the protein sequence has similar functional domains, it may represent evolutionarily related

[8, 9]. If there is a similar functional domain in the protein sequence, it may be evolutionarily related."

"Comparing similarities with RPS-BLAST through the Conserved Domains database (CDD) [10]."

"Proteins of different functions, sources and species, in addition to structural information verification through time-consuming experimental methods,if the homology between the sequences is low, it is more difficult to find the protein aggregation rules by using the alignment method."

......

Response:

Thank you for your advice, and we appreciate the chance to make this manuscript clear. We have our manuscripts checked by English language native speakers for English editing. The correctness of English writing has been improved. In addition, the English revision certificate has been uploaded as an attachment.

- "The quaternary structure of the homo-oligomer can be encoded by the Equation an, where n is the copy number of the polypeptide chain." This sentence has been deleted because of its inappropriateness.
- "If the protein sequence has similar functional domains, it may represent evolutionarily related [8, 9]. If there is a similar functional domain in the protein sequence, it may be evolutionarily related." These sentences have been re-written to “If the protein sequence has similar functional domains, it may represent an evolutionarily relationship [8, 9].”, and deleted another inappropriate sentence.
- "Comparing similarities with RPS-BLAST through the Conserved Domains database (CDD) [10]." This sentences has been re-written to “Comparing similarities can be performed with RPS-BLAST through the Conserved Domain Database (CDD) [10].”
- "Proteins of different functions, sources and species, in addition to structural information verification through time-consuming experimental methods,if the homology between the sequences is low, it is more difficult to find the protein aggregation rules by using the alignment method." This sentences has been re-written to “For proteins of different functions, sources and species, in addition to verifying structural information, it is more difficult to find the protein aggregation rules using the alignment method if the homology between the sequences is low.”
