# Supplementary material for: QUATgo: Protein quaternary structural attributes predicted by two-stage machine learning approaches with heterogeneous feature encoding
Source: PLoS One. 2020 Apr 29;15(4):e0232087. doi: 10.1371/journal.pone.0232087 (PMC7190164; doi:10.1371/journal.pone.0232087)
Supplement: Attachment — Submitted filename: Response to Reviewer_QUATgo_0323_v2.docx [file pone.0232087.s002.docx]

**Institute of Genomics and Bioinformatics**

**National Chung Hsing University**

**Taichung, Taiwan 40227 ROC**

March 23, 2020

Dr. Mingyang Lu

Academic Editor

PLOS ONE

Dear Dr. Lu,

Thanks for your careful review of my manuscript (PONE-D-19-21078). I really appreciate your help and the comments from both reviewers. After carefully reading the comments raised by the reviewers, I think most comments or problems can be solved. After trying our best to answer all the comments, I would like to resubmit this revised manuscript. All the revised portions were highlighted in yellow color in this revised manuscript. Many thanks for your help. Looking forward to hearing from you.

Sincerely yours,

Yen-Wei Chu

Professor

Institute of Genomics and Bioinformatics

National Chung Hsing University

Taichung, Taiwan 40227 ROC

Tel: 886-4-22840338 ext. 7041

Fax:886-4-22859329

E-mail: ywchu@nchu.edu.tw

**I. Answer to comments from Reviewer #1:**

I carefully read the revised manuscript and the response to all reviewer concerns. I think the authors successfully responded to my concerns and recommend now acceptance of the manuscript.

Response:

Thank you for your advice.

**II. Answer to comments from Reviewer #2:**

A major issue with the MS is the error in MCC calculation of their QUATgo in Table 9. Based on a quick check on Homo-12-mer, QuarIdent has a much larger precision (0.939 vs. 0.271), close specificity (0.998 vs. 0.999), and slightly worse sensitivity (0.018 vs. 0.030), yet its MCC value is much worse than that of QUATgo (0.067 vs. 0.990 !). This does not make sense. In fact, based on the values of precision, sn, sp, I can infer its MCC value is 0.0866846 that is slightly better than 0.067 of QuarIdent. Many other MCC values are also wrong, i.e. Monomer: MCC=-0.103 instead of 0.378;Homo-2:MCC=-0.123; Homo-3: MCC=-0.182 ... On average, their MCC value is worse than that of QuarIdent ! Unless the authors correctly calculate their MCC and show they are statistically significantly better than other methods (i.e. p-value < 0.05 by student-t test), I do not see the value of publishing this work.

Response:

Thank you very much for your suggestions. We found that there was a bias towards negative in the QUATgo system. It shows that the specificity (sp) was exceedingly high and the sensitivity (sn) was too low. Therefore, we spent an extra three months to re-adjust the test set and revise it to the ratio of positive and negative set equal to 1:1. Finally, accuracy and reliability are improved, and the results will not be excessively biased towards sp. Due to the update of the test data set, there are many corrections in the table and the article, which we have highlighted in yellow. Besides, we modify the cases discussed in the case study. We used the Influenza hemagglutinin protein as our case study. The results showed that 8 of 13 hemagglutinin proteins correctly predicted their quaternary structure (hetero 6-mer), while the QuatIdent and QuaBingo were all wrong. Moreover, the sequence identity between these 13 hemagglutinins and our train data set is less than 40%. Thanks for your reminding, let us be able to improve and make the research results more precise.
